# Edge‐detection of the radiation field in double exposure portal images using a curve propagation algorithm

**DOI:** 10.1120/jacmp.v9i4.2710

**Published:** 2008-07-02

**Authors:** Patricia Lasserre, Bryce Cutt, James Moffat

**Affiliations:** ^1^ Irving K. Barber School of Arts and Sciences University of British Columbia Okanagan Kelowna Canada

**Keywords:** double portal imaging, segmentation, thresholding, fast marching method, edge localization

## Abstract

An accurate detection of the radiation field is crucial to 3D conformal radiotherapy (3D‐CRT). Automated techniques to detect the field edges on double exposure portal images have previously focused on thresholding techniques. In this paper, we present a new approach based on a curve propagation technique (the Fast Marching method) which proves to be more effective at detecting the radiation field than its thresholding counterpart. The comparison of both techniques in terms of computational speed and effectiveness of the detection is presented using complex images with non‐homogeneous intensity levels inside the radiation field, and gradual variations in intensity level at the field boundaries. Results show that our Fast Marching method is easier to automate, and converges faster to the boundaries of the segmented radiation field. The computation time of the Fast Marching technique is five times faster in typical portal images.

PACS numbers: 87.53.Oq, 87.57.Nk, 87.57.‐s.

## I. INTRODUCTION

In three‐dimensional conformal radiotherapy (3D‐CRT), multiple high‐energy radiation beams are delivered to tumors to kill cancer cells. The portal verification of the positioning of these radiation fields is carried out by comparing the location of the treated portal image taken during delivery with the reference simulation, which is an image taken on a simulator or a digitally reconstructed radiograph (DRR).^(^ ^1^ ^–^ ^5^ ^)^ Developing an efficient method for an automated verification of the treatment portal localization is crucial to the quality assurance of conformal radiotherapy.^(6)^ ^,^ ^(7)^ If localization accuracy is improved, it is possible to envision applying higher dosage to the tumor with exclusion of excess to normal tissues.

Previous investigators have tested many of the edge‐detection techniques traditionally used in computer vision to determine the boundaries of the radiation field. A two‐step process combining histogram thresholding and Sobel gradient operator was applied by Gilhuijs and Van Herk on portal images.^(2)^ ^,^ ^(5)^ Leszczynski et al.^(6)^ indicated that the first derivative of a Gaussian (DoG) was the best option for step‐like edges in images corrupted by noise. Wang et al.^(8)^ suggested that the morphological gradient^(9)^ was a valid alternative to DoG: where the gradient images were similar, the computation time was reduced. Petrascu et al.^(10)^ offered an original approach of multi‐scale edge detection with wavelets for both the radiation field and the anatomical structure. The wavelets segmentation approach, in combination with a deformable model has also been used by Burnett et al.^(11)^ for the demarcation of the spinal canal on CT images. Many segmentation techniques such as the watershed segmentation,^(12)^ ^,^ ^(13)^ graph theory‐based segmentation,^(14)^ and deformable models, have also been extensively applied in other areas of medical imaging. Interestingly, the idea of a deformable model, while natural, has not been tested for this particular problem. The popular Fast Marching and Level Set methods^(15)^ have been used in a variety of medical images^(^ ^11^ ^,^ ^16^ ^–^ ^20^ ^)^ because of their ability to spatially constrain the boundaries of a region. Humnabadkar et al.^(17)^ illustrated that many biology applications could potentially benefit from using these types of techniques in X‐ray images of lungs and pathological images of blood cells. Level Set methods are especially usable when prior shape knowledge is available. Known as Level Set shape prior, Rousson et al.^(19)^ proposed to strongly constrain the contour to follow a known shape. Their algorithms compensate for the disappearance of the contour shape in images by relying on the known data. Tsai et al.^(21)^ used prior shape variation knowledge to deduce the initial contour for the Level Set method. To minimize the influence of noise, they used an energy function based on global characteristics of the image instead of the local gradient information.

Thresholding techniques associated with gradient information provide adequate segmentation results in images with homogeneous density. However, these techniques have two major drawbacks. First, their computation time gets quite high as soon as the image size and resolution increase. This paper presents a modification of the automatic detection of the threshold proposed by Wang et al.^(8)^ to significantly reduce the computation time. Second, they fail to find the correct edges of the radiation field when the intensity variation is weaker on the boundaries of the radiation field than inside the treated area. This failure situation arises when the tumor is located in areas where the density varies dramatically, or when the radiation field is wider than the body anatomy area. Typical clinical situations include treatment portals of the head, neck, and chest, or hip area with a metallic replacement. In order to provide a fully automated system, extreme situations such as these should be accommodated with limited operator intervention. We also present an enhanced version of the gradient which includes ‘a priori’ information to remove additional noise, which suggests that the Fast Marching method has the potential to be successful in detecting radiation field on a large variety of images, from simple to complex. In addition, the paper presents the result of the comparative analysis of the traditional thresholding technique with the Fast Marching method. We compare edge detection results and computational times for both the traditional thresholding technique and its enhanced version of the gradient, with the Fast Marching technique. We conclude that the Fast Marching technique is more efficient both computationally and qualitatively, and has more automation potential.

## II. METHODS AND MATERIALS

### A. Materials

Tests were conducted on an Athlon 64 3700+machine. The software was written in Java (version 1.5.0) and integrated as a set of image operations in an open source software package (the Java Vision Toolkit 2). Testing of different segmentation methods was carried out on double exposure portal images from a variety of anatomical sites (head and neck, pelvic areas, chest, etc.). These gray‐scale images were taken from two different machines, the Elekta SL 20 linear accelerator equipped with an iView GT flat panel digital imager (16‐bit and 1024×768 pixels) and the SL‐75 accelerator equipped with a video analog imager (8‐bit and 768×576 pixels). A total of 65 images were selected to represent an adequate sample of portal complexities encountered for each anatomical site, with a bias toward more complex images than homogeneous ones. Of the 65 images selected, 32 were considered highly complex images, 16 of which were for the head and neck, 4 for the hip, and 12 for the lungs. A total of 14 images were considered to be of a medium complexity including 6 for the head and neck, 2 for the hip area, and 6 for the lungs. The remaining images were considered to be of low complexity.

**Figure 1:**
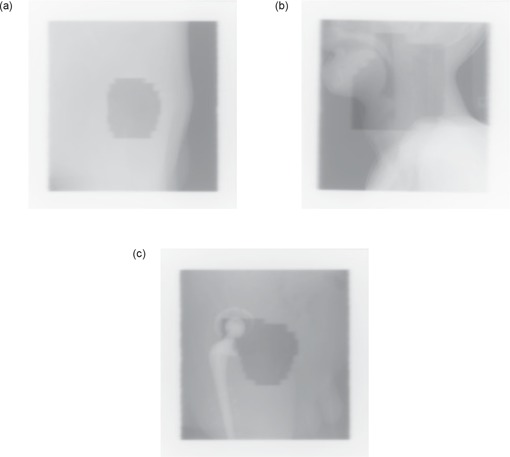
Sample images: (a) A simple image where edges can easily be detected. (b) Complex image due to internal gray‐level variations. (c) Complex image of the radiation field applied to a patient who had a hip replacement.

An image was deemed complex if it had features that confounded standard segmentation methods. For instance, strong edges inside the image area to be segmented caused the methods to over‐segment the image. Another feature was weak or gradual edges on the boundary of the analyzed area such that the methods did not distinguish the edge from the noise within the image, causing under‐segmentation. Fig. 1 illustrates classical examples of images of various degrees of complexity.

### B. Thresholding of gradient

The standard edge‐detection algorithm used to detect the radiation field on the portal images was challenged by the image size and intensity resolution. As proposed by Wang et al.,^(8)^ ^,^ ^(22)^ it automatically detected the optimal threshold on the gradient by sequentially testing the results for each thresholding. The technique consisted of counting the number of large areas for a specific threshold, then considering the optimal threshold as the one for which the number of large areas is greater than 2 (one being the radiation field). The initial value of the threshold, the largest gradient value in the image, dropped down until the condition was reached. This method became computationally intense due to the large number of intensity levels on some of our images. We subsequently improved the method as follows:

#### 1. Improvement of the automatic optimal threshold search

A typical technique to reduce computational time is to replace a linear search with a binary search. The idea is to jump the threshold value up or down based on the number of large regions detected for that threshold. If the number of large regions is smaller, the threshold is then reduced, else the threshold is increased. The binary search has the advantage of giving a ${\text{Nlog}}_{2}$N upper bound in the number of iterations required for the search; N is the number of gray level values to search through. There is a maximum of 16 iterations for a 65,536 gray level 16‐bit image. The number can further be reduced by observing that optimal thresholds are never above a certain level and starting the binary search from that point on.

#### 2. Directional gradient

Both DoG (derivative of Gaussian) and morphological gradient thresholding were tested. In both cases, the noise level after calculation of the gradient was still very high. Application of a Gaussian filter could compromise the accuracy of the edge detection but more importantly, attenuated even more some of the weakest intensity areas at the boundary. Many pre‐processes such as the conversion of images to 8‐bit images and the use of adaptive thresholding were unsuccessful on our test images. The level of noise was as high as the level of the weak intensity edges, making it impossible to distinguish one from the other. To remove some of the noise without altering the initial boundaries information of the image, we made ‘a priori’ assumptions about the process as discussed in Wang et al.^(8)^

It is reasonable to assume that the radiation field is centered in the image. Considering the nature of portal images, it is also natural to consider that the edge of the shape of interest is always a transition from darker to lighter shades of gray in the direction away from the center of the shape. The purpose of the directional gradient is to filter from the image all gradient values which are not in the direction of interest from the center point. This directional information is obtained by calculating the vector dot product of the gradient and the vector joining the pixel value to the central point. (A positive result indicates both vectors point to the same direction). As the shape of the radiation field becomes more complex (concavities, strong variations inside the radiation field), several center points may be required to improve the results (the dot product is then performed using the closest point).

### C. Fast Marching method

An elaborate technique that has been extensively studied in the last ten years is the active contours method, also called snakes or parametric contours.^(23)^ ^,^ ^(24)^ Since its first appearance in Kass et al.,^(25)^ this technique has given birth to new algorithms based on the theory of curve and surface evolutions, such as Level Set and Fast Marching methods.^(15)^ ^,^ ^(26)^ ^,^ ^(27)^ The idea behind the Fast Marching and Level Set methods is to represent the segmentation process as a propagating curve on which forces are applied. The forces applied can take both local and global information into account. The motion of the curve is described with a partial differential equation, which is solved using conservation laws. As described in Sethian's book,^(15)^ the Fast Marching method consists of solving the Eikonal equation:

(1) |∇T|F=1,  T=0 on τ

where T is the arrival surface, F is the force applied (always positive), and τ is the initial location of the front. The Level Set method, which consists of solving a Hamilton‐Jacobi equation:

(2) $$\vartheta_{t}+F|\nabla \vartheta |=0,where\;\tau (t)=\{(x,y)|\vartheta (x,y,t)=0\}$$

is the propagation front. The level function $\nu_{\mathrm{03BD}}$ is a function based on the arrival surface T, which takes into account the fact that the force F can now be arbitrary. This equation solves the more general problem of having the curve either expand or contract at any given time whereas the Fast Marching method constrains the movement of the curve to one direction only (expanding or contracting) by enforcing F to be positive. Computationally, the Level Set method is much more intense and is often used after an initial contour has been located close to the boundaries to be detected.

The result from the directional gradient indicated that the active contour approach is an interesting alternative. While we had prior knowledge of the shape applied, it could not be used to constrain the shape of the resulting contour if quality assurance on the radiation field being applied was to be performed. For this reason, our focus was on implementing the Fast Marching method and not on prior shape segmentation.

Details on the Fast Marching method algorithm can be found on Q. Lin's implementation.^(28)^ The force applied is inversely proportional to the gradient image as described in Sethian's book:^(15)^

(3) $$F=g_{I}(x)=\frac{1}{1+|\nabla (G_{\sigma }*I(x,y))|}$$

where the expression $G_{\sigma }^{*}I$ denotes the image *I* convolved with a Gaussian smoothing filter. As the gradient values are close to zero (no changes in the image), the force applied tends to 1, moving the curve; on the contrary, where large changes occur, the gradient value is large, and F tends to zero, forcing the curve to remain ‘stable’ at its location. It is important to note that the force F does not ‘stop’ the curve movement, but slows it for large gradients. Should the number of iterations be large enough, the curve would propagate up to the limits of the image, jumping over any edge transitions.

The force we applied on our image set was a variation on Equation (3). Smoothing the image with a 5×5 Gaussian filter before running the Fast Marching method filtered out some of the internal noise but also smoothed the edge transitions that we wished to detect. To accentuate gradient transitions that had been reduced by the application of the Gaussian filter, the gradient term described in Equation (3) was squared:

(4) $$G_{\sigma }=\frac{1}{273}\left[\begin{array}{ccccc} 1 & 4 & 7 & 4 & 1\\ 4 & 16 & 26 & 16 & 4\\ 7 & 26 & 41 & 26 & 7\\ 4 & 16 & 26 & 16 & 4\\ 1 & 4 & 7 & 4 & 1 \end{array}\right]$$

(5) $$F=g_{I}(x)=\frac{1}{1+(\nabla (G_{\sigma }*I(x,y)))}$$

This implies that the larger the gradient value, the faster the force F decreases. The effect on the curve propagation was to reduce even more the speed at which the curve moved in regions with strong gradient values, allowing the curve to continue moving in regions with small gradient values. In our image set, the edge was frequently well defined in one or several areas, but more diffuse elsewhere. Slowing down the curve on the defined areas allowed the algorithm to detect the less defined portion of the boundaries without having the curve pass over the strong edges. (Remember that the curve is slowed but not stopped. The stopping is fixed by the number of iterations selected.)

## III. RESULTS

### A. Automatic thresholding technique

#### 1. Binary vs. linear automatic threshold search

The binary search method generally worked well. Unfortunately, the binary search test to determine if the threshold should be increased or decreased was not reliable. The assumption for a binary search to be successful is that the data set be sorted. The algorithm relies on a continuous increase in the number of large regions as the threshold is lowered. This was not always the case. For example, it was possible to have a threshold for which three large regions were found in between two threshold values for which there was a lower number of regions. In fact, the threshold provided using the binary search did not always correspond to the optimal threshold satisfying the condition as proposed in Wang et al.^(8)^ However, tests on our image set revealed that when a good threshold was found, the same value was obtained by the linear search and the binary search. The binary search failed to provide results on 10 images out of the 65 tested but this did not justify rejecting its computational improvement. When the two methods disagreed on the values of the optimal threshold, neither value was a good threshold. It meant that both methods were unsuccessful at correctly segmenting the radiation field. It was also interesting that when the two search methods found the same threshold, they did not miss a better threshold in the image.

#### 2. Morphological gradient vs. directional gradient

**Figure 2:**
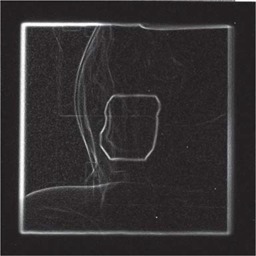
Morphological Gradient.

**Figure 3:**
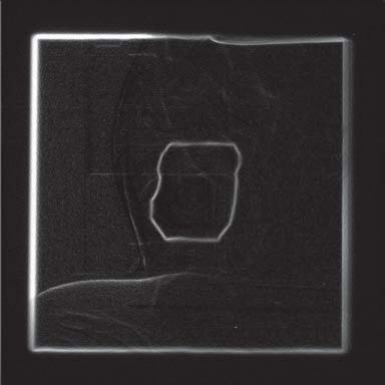
Directional Gradient.

Figs. 2 and 3 show the difference between the morphological and the directional gradients.

In Fig. 3, most of the strong gradient values due to the body anatomical boundaries have disappeared. Most importantly, the internal variations inside the radiation field area have also been eliminated, thus greatly facilitating the detection of the edges of the radiation field. This method is efficient at filtering contextual noise, and improving the quality of the gradient image; only the gradient values of potential interest for the detection of the radiation field edges are left in the image.

**Figure 4:**
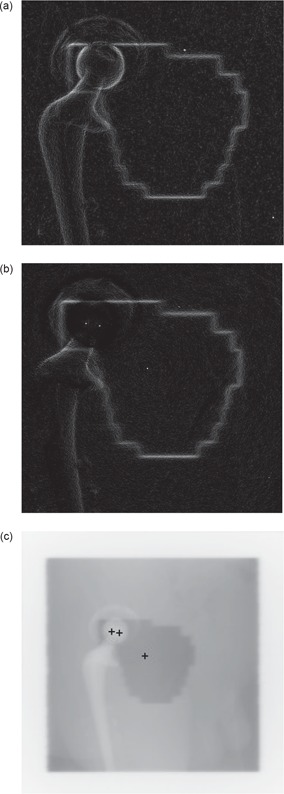
Morphological vs. Directional Gradient: (a) Morphological gradient; (b) Directional gradient (3 initial points); (c) Location of the 3 initial points used for the directional gradient.

Fig. 4 presents the results of the gradient using the morphological method and the directional method (resulting images have been cropped to display only the area of interest). As previously shown, the directional gradient eliminates strong gradient values that do not pertain to the radiation field edge. The previously weak transition close to the hip replacement is now amplified.

Fig. 4(c) shows the location of the three initial seed points that the directional gradient relies on. The method is sensitive to the choice of these seed points. Two were chosen in the hip replacement area to reduce the effects of its internal variations and to ensure all angles of view were explored. Almost the entire bone structure was eliminated while keeping the weak gradient values of the radiation field. Choosing points too close to each other may not produce the desired effect as the closest point is used to determine whether to filter the gradient, and might not be appropriate. The balance between the number of points chosen and their positioning is critical, and when done manually for each image, makes the method less attractive for complex images. In Fig. 4 for example, it took more time to manually find those three effective points than it would have taken an operator to draw a better outline by hand.

### B. The Fast Marching method

The Fast Marching method was applied on our image set with several initial fronts positioned at center point (one seed point only), at several locations inside the radiation field area (several seed points), and on an initial contour.

**Figure 5:**
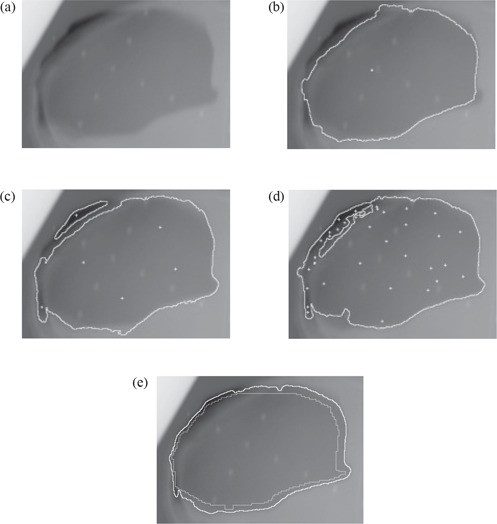
Fast Marching method: (a) Original image; (b) Result with one central point; (c) Result with 4 points in homogeneous area, 2 in dark areas; (d) Result with more than 10 points; (e) Result (white outline) with an initial contour (light gray outline).

The first example, Fig. 5, shows the result of the Fast Marching method using different initial starting fronts.

Fig. 5(b) shows that the method worked well for the large homogeneous part of the radiation field but did not moved into the darker areas on the top left and bottom left of the radiation field. The second example, Fig. 5(c), used multiple starting points with one seed point in each of the dark areas and four seed points in the homogeneous area. One can notice that there is too much change in pixel value, in and around the dark areas, for the Fast Marching method to join the multiple shapes. Fig. 5(d) demonstrates what happens when more starting points are scattered in areas where the previous initial points were ineffective. While the scatter improved the results, it took many carefully placed points to obtain satisfactory results. Starting from a center point was ideal, but not realistic as the images became extremely complex. It worked well when the radiation field area was relatively homogeneous. Selecting several starting points was efficient if the radiation field consisted of a number of separate shapes in different parts of the image, or the field was all in one area of the image but had multiple distinct radiation levels (gray‐scale levels) that needed be joined together. The most efficient technique was to use a proportionally reduced version of the initial shape of the radiation field as initial contour (Fig. 5(e). In fact, this method was successful in detecting the right radiation field areas in all images tested because most of the internal variations were completely avoided. To automate the process as much as possible, the initial contour was generated automatically from the reference simulation and then scaled to be inside the radiation field using a series of stepping erosions. An attractive aspect was that, when using the initial outline generated from the reference simulation, the threshold used to stop the Fast Marching method was identical for all images of the same size (one threshold for 16‐bit images, one threshold for 8‐bit images). When starting from single or multiple points, no unique threshold worked in all cases.

## IV. DISCUSSION

We compared the automatic thresholding technique and the Fast Marching method both in terms of quality of segmentation and computational speed. We previously mentioned that the results of the linear and binary search were equivalent. So, we limited the computational time evaluation to the binary search only (the linear search being obviously more time consuming). The Fast Marching method initialized by a contour was successful, even in the difficult situations of complex portal images. The automatic thresholding technique, even in relatively simple images, could be disturbed by noise, creating some small regions that required additional processing. On the contrary, the Fast Marching method ignored those details to focus only on the expected boundaries.

**Figure 6:**
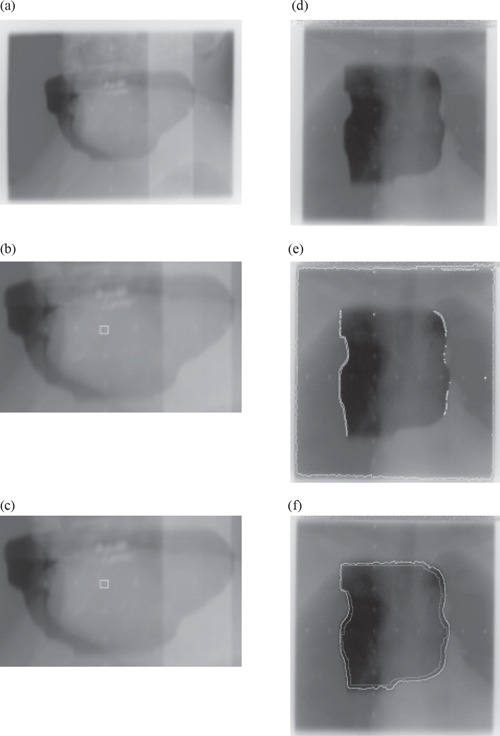
Comparison Thresholding vs. Fast Marching method (FMM): (a) Original 8‐bit image; (b) Thresholding technique – C.P.U. time=4.5 s; (c) FMM (initial contour inside) – C.P.U. Time=0.8 s; (d) Original 8‐bit image; (e) Thresholding technique – C.P.U. time=2.9 s; (f) FMM (initial contour inside) – C.P.U. time=0.9s.

Fig. 6 illustrates the difference between the two methods. To visualize the result better, the original image and its segmentation, using the thresholding technique and the Fast Marching method, are presented in one column. The segmented images are cropped to display the area of interest only. However, the segmentation was done on the entire image. Figs. 6(a) to 6(f) present some cases for which the thresholding technique failed for two reasons. In Figs 6(a) to (c), no valid threshold could be detected through binary nor linear search. Whereas in Figs. 6(d)) to 6(f), the threshold was too low, yielding an under‐segmentation of the image as indicated by the white outline around the image border. However, in both cases, the Fast Marching method determined the outline quite well. It is important to note that computational time was saved when using the Fast Marching method, both on 8‐bit and 16‐bit images.

Table 1 compares the computational times and image segmentation quality. The computational time includes all the pre‐processes and the segmentation itself. Results on the 16‐bit images only (25 images) are presented for the thresholding technique as it was not applicable to 8‐bit images due to a non‐consistent set of assumptions and a different number of large regions. Because the Fast Marching method is more dependent on the size of the surface to propagate and the number of iterations to reach the contour than on the image itself, the difference between 8‐bit and 16‐bit images is not high (roughly 1 second). However, there is a time factor of five between the two methods, making the Fast Marching method more practical in a working environment.

**Table 1:** Comparative results (computational/qualitative)

*Segmentation Method*	*Segmentation Results % ACCEPTABLE*			*C.P.U. time (in seconds)*		
	*easy*	*medium*	*complex*	*easy*	*medium*	*complex*
Automatic thresholding (binary) 16‐bit images (25 in total)	18/18 (100%)	2/4 (50%)	0/3 (0%)	10.8	10.9	13.3
Fast Marching Method	19/19 (100%)	14/14 (100%)	31/32 (96.8%)		0.9 (8‐bit images) 2.0 (16‐bit images)	

Qualitatively, the segmentation result was deemed acceptable when the detected boundaries were close to what was visually expected. The thresholding technique failed as soon as images were complex. The Fast Marching method results were attractive considering that only one image was deemed unacceptable. From the start, this image was considered the ‘ultimate’ difficulty even for the human eye. Both in terms of segmentation results and computational times, the Fast Marching method was much more robust in the context of an automated process to detect radiation fields.

The results were consistent with previous local and global segmentation results. The thresholding technique depended on global information to segment the image. It was efficient when the regions of interest were clearly defined (i.e., homogeneous regions) because an effective threshold could separate the different regions in the image. However, when density variations in those regions were more important than the variations between regions, that technique could not correctly identify a threshold. In contrast, the Fast Marching method relied on local gradient information to determine where the next position of the curve was. Therefore, as demonstrated when using the initial contour close to the boundaries, it was not perturbed by the density variations inside the treated area. However, the Fast Marching method was more sensitive to those internal density variations when the initial front consisted of scattered points; more of the inside of the treated area was used as local data to determine the next movement of the curve, re‐introducing some of the global information problem encountered by the thresholding technique.

Currently the Fast Marching method uses the magnitude of the gradient to help calculate how fast it can get to specific points of the image as it propagates, with no regard to the direction of the gradient. However, the Fast Marching method takes some directional information into account by limiting the direction in which the curve can move. Incorporating the idea introduced in the directional morphological gradient method might bring better accuracy to the results. As currently done, though, it would mean losing the benefit of the Fast Marching method being automated.

The Fast Marching method is a special case of the Level Set method in which the interface is only able to move in one direction. It is much faster computationally but the Level Set method could be far more effective when properly constrained as it expands and contracts its interface at different times in different parts of the interface as needed by the image. In this particular application, the benefit of the Level Set method is unlikely to overcome its increased running time. A combination of the Fast Marching method with Level Set method would be more attractive for real‐time follow up of contours. This technique would be interesting to test for the detection and follow‐up in real‐time of gold seed markers as used to locate prostate in radiotherapy portals.^(27)^

## V. CONCLUSION

We presented an effective approach in detecting radiation fields in doubly exposed portal images based on a curve propagation technique (the Fast Marching method). This approach proved to be more effective at detecting the radiation field than its thresholding counterpart, even with an improved gradient which took directional context into account. The comparison of both techniques in terms of computational speed and effectiveness of the detection showed that our Fast Marching method was easier to automate, and converged faster to the boundaries of the segmented radiation field. The computation time of the Fast Marching technique was five times faster in typical portal images.

The results could be further investigated as follows. The Fast Sweeping method ^(^ ^29^ ^,^ ^30^ ^)^ provides an alternative to the more complex algorithm required in the Fast Marching method by eliminating the need for a heap structure. Hua Li et al.,^(20)^ for example, applied the Fast Sweeping method to their 2D boundary tracking algorithm to ensure computational efficiency. We would like to compare the performance and qualitative results of the Fast Sweeping method with the Fast Marching method.

## ACKNOWLEDGMENTS

This work was supported by the Grant‐In‐Aid program of Okanagan University College, and the UBC Okanagan Internal Research Grant Support program.

The authors are grateful to Dr. Yves Lucet for his assistance, and are indebted to Dr. Rasika Rajapakshe from the BC Cancer Agency in Kelowna who provided all the images along with his medical expertise.
